# Management of Anterior Skull Base Defect Depending on Its Size and Location

**DOI:** 10.1155/2014/346873

**Published:** 2014-05-07

**Authors:** Manuel Bernal-Sprekelsen, Elena Rioja, Joaquim Enseñat, Karla Enriquez, Liza Viscovich, Freddy Enrique Agredo-Lemos, Isam Alobid

**Affiliations:** ^1^Department of ORL-HNS, Hospital Clinic, University of Barcelona Medical School, 08036 Barcelona, Spain; ^2^Department of ORL-HNS, Rhinology and Skull Base Unit, Hospital Clínic, University of Barcelona Medical School, 08036 Barcelona, Spain; ^3^Department of ORL-HNS, Althaia Xarxa Assistencial de Manresa, 08243 Manresa, Spain; ^4^Department of Neurosurgery, Hospital Clínic de Barcelona, 08036 Barcelona, Spain; ^5^Universidad del Valle, 76000 Cali, Colombia

## Abstract

*Introduction*. We present our experience in the reconstruction of these leaks depending on their size and location. *Material and Methods*. Fifty-four patients who underwent advanced skull base surgery (large defects, >20 mm) and 62 patients with CSF leaks of different origin (small, 2–10 mm, and midsize, 11–20 mm, defects) were included in the retrospective study. Large defects were reconstructed with a nasoseptal pedicled flap positioned on fat and fascia lata. In small and midsized leaks. Fascia lata in an *underlay* position was used for its reconstruction covered with mucoperiosteum of either the middle or the inferior turbinate. *Results*. The most frequent etiology for small and midsized defects was spontaneous (48.4%), followed by trauma (24.2%), iatrogenic (5%). The success rate after the first surgical reconstruction was 91% and 98% in large skull base defects and small/midsized, respectively. Rescue surgery achieved 100%. *Conclusions*. Endoscopic surgery for any type of skull base defect is the gold standard. The size of the defects does not seem to play a significant role in the success rate. Fascia lata and mucoperiosteum of the turbinate allow a two-layer reconstruction of small and midsized defects. For larger skull base defects, a combination of fat, fascia lata, and nasoseptal pedicled flaps provides a successful reconstruction.

## 1. Introduction

Cerebrospinal fluid (CSF) leaks may be continuous or intermittent. Dura and arachnoid membranes need to be interrupted and usually there is a bony defect too. Patients with a skull base defect are at risk of suffering ascending bacterial meningitis by over 10% per year, independently of the size or location of that defect [1, 2]. Intermittent leakage may be difficult to assess. Different reconstructive techniques have been described to close skull base defects and termed “onlay,” “overlay,” “underlay,” and “inlay” procedures. Also, different materials, mainly autologous, have been used for the reconstruction. All of them seem to work well.

Among frequent symptoms one may find a watery rhinorrhea, mainly unilateral, and sometimes headaches when the fistula is associated with a meningocele or ascending meningitis [3]. The most frequent location of the leak is the cribriform plate followed by ethmoidal roof, sphenoid, frontal sinus, sella turcica, and clivus [1].

With the advent of extended endoscopic skull base resections, the need for large reconstructions has increased, including those of high pressure/high flow leaks communicating with the 3rd ventricle. For these CSF leaks the recurrence rate was above 30%, questioning the advantages of expanded endoscopic skull base approaches. Since the description of the pedicled nasoseptal flap [4] the incidence of postoperative CSF leaks could be considerably reduced down from 33% to 5.4% [5].

The goal of our retrospective study is to evaluate the rate of effective closure of our skull base defects depending on their size and location in order to establish an algorithm for the diagnosis and treatment.

## 2. Material and Methods

Patients that underwent an endoscopic skull base reconstruction between February 1998 and January 2013 were included. A retrospective chart review was performed to assess causes, location, type of presentation, preoperative studies, intraoperative findings, and surgical technique of reconstruction.

Patients were divided into two groups. 

*Group 1*. Patients undergoing expanded endoscopic skull base surgery for intracranial pathology between 2007 and 2013 were included (large skull base defects, >20 mm). Patients undergoing pituitary surgery were excluded from evaluation.

**Figure 1:**
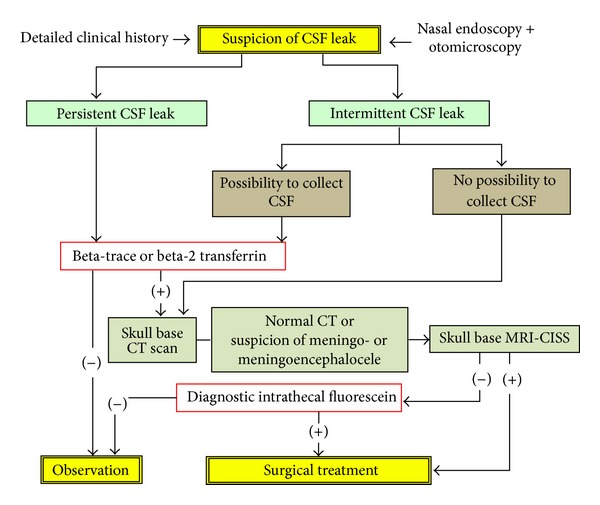
Algorithm of the management of CSF leaks. CISS = constructive interference in steady state.

*Group 2*. Patients with rhinoliquorrhea of other origins operated on between 1998 and 2013 were included (small defects, between 2 and 10 mm, and medium-sized defects ranging between 11 and 20 mm). For the diagnosis and management of these cases a protocol has been established (Figure 1).

In both groups antibiotic prophylaxis consisted in intravenous administration of ceftriaxone for 5–7 days. In allergic patients levofloxacin and trimethoprim/sulfamethoxazole were considered appropriate alternatives.

Group 1 undergoing expanded skull base surgery included 54 patients suffering from different tumors involving the skull base. All patients underwent CAT scan and MRI.

### 2.1. Technique of Surgical Reconstruction for Group 1

**Figure 2:**
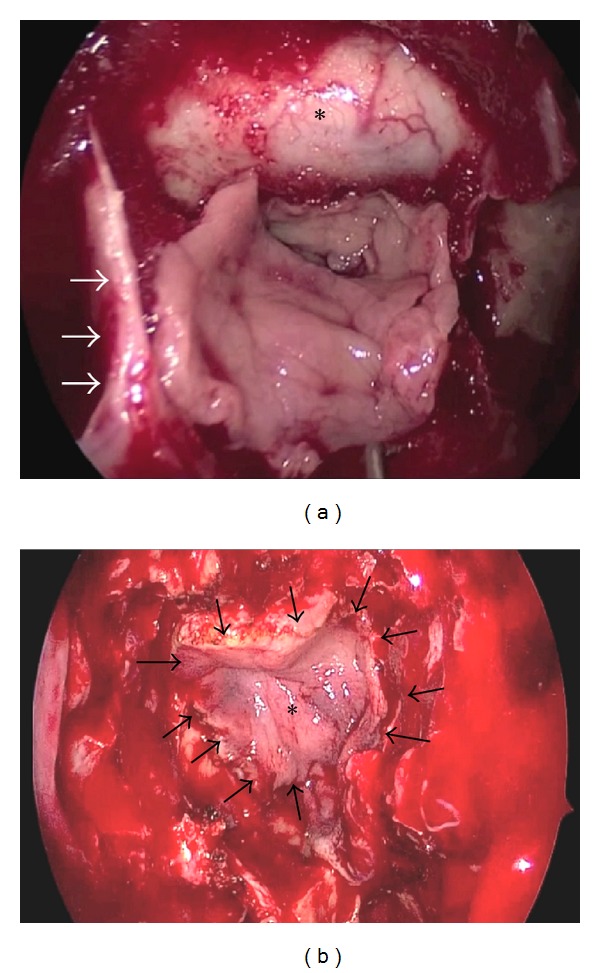
(a) Nasoseptal pedicled flap dissected from the left side. Arrows pointing to remnant of the inferior aspect of the vomer and *dura. (b) Nasoseptal flap (∗) positioned over the skull base. Arrow pointing at the edges of the flap.

Lumbar drainage was carried out before starting the endoscopic surgery and harvesting of suprapubic fat, particularly in cases in which a high flow CSF leak was expected. A pedicled nasoseptal flap was created [4]. Side and size of the flap depended on the calculated defect. After tumor removal, the suprapubic fat served to fill dead spaces between the brain and the dura. The fat was then covered with lyophilized fascia lata (TSF, Barcelona, Spain) in an “*underlay*” position, that is, between the fat and on the bone of the remnant skull base in the epidural space. The nasoseptal flap covered the bony borders of the defect and was fixed either with blood and Surgicel*©* or with fibrin glue, the latter not being mandatory. A finger cot packing was used to avoid adherences (Figures 2(a) and 2(b)).

Patients stayed in bed for 72 h with an antithrombotic prophylaxis. Blowing the nose or sneezing with open mouth was forbidden. In order to avoid increased abdominal pressure diet rich in fibers was provided and sometimes a laxative was prescribed. Packing was withdrawn 24–48 hours after surgery, and the lumbar drainage took place after 72–96 hours in case no sign of an active fistula was observed. In case of suspicion of a CSF leak, intrathecal fluorescein application through the lumbar drainage was performed as a diagnostic procedure. CAT scan or MRI was scheduled after 24 hours to rule out intracranial bleeding or pneumoencephalus.

### 2.2. Technique of Surgical Reconstruction for Group 2

Group 2 underwent a more limited endoscopic revision of mainly the anterior skull base. All patients with a spontaneous CSF leak were submitted to an ophthalmologic exploration in order to rule out benign endocranial hypertension. All patients underwent a CAT scan of the paranasal sinuses and anterior skull base in 1 mm slices, which allowed assessment of the skull base defect preoperatively. In cases with suspicion of a meningocele or meningoencephalocele an additional cranial MRI was performed. Biochemical conformation of CSF was performed by means of beta-2 transferrin until 2005, and after that period the beta-trace protein kit was introduced. Measurement of the skull base defect was accomplished with the help of the CAT scans and, intraoperatively, with the branches of a 45° Blakesley forceps on the defect.

**Figure 3:**
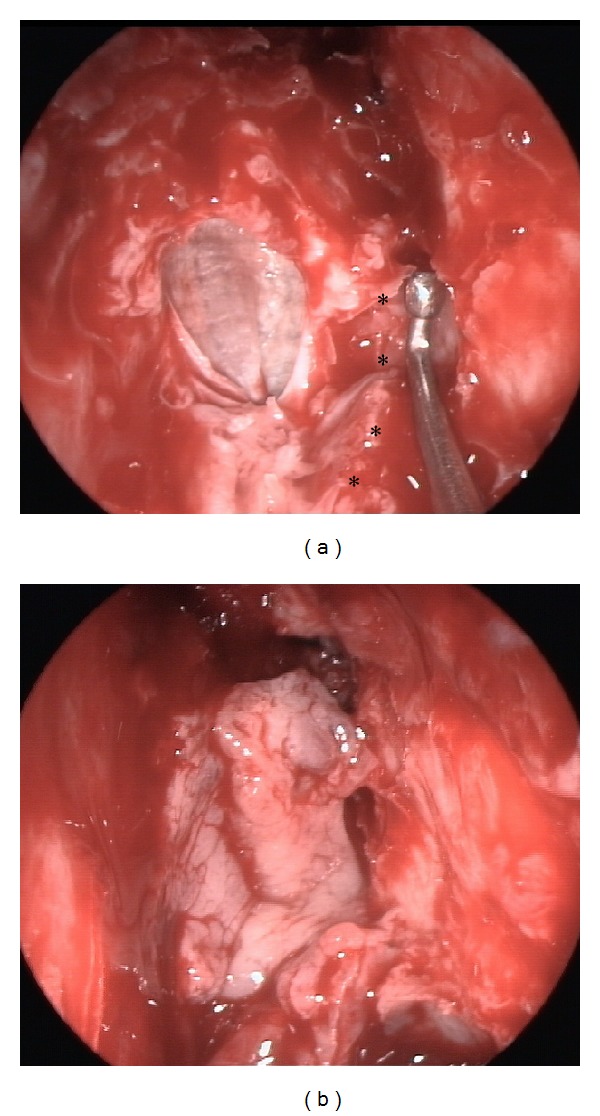
Reconstruction of a defect at the left cribriform plate. (a) Fascia lata introduced in an overlay position. Intracranial pressure and pulsations end up pushing the fascia onto the dura. Middle turbinate resected (∗ indicating its original attachment). (b) Free mucosal graft from the middle turbinate.

Around 1 hour before surgery 0.5–1 mL of 5% sodium fluorescein was applied intrathecally. Dilution was performed with distilled water. Fluorescein was found to be helpful in identifying the skull base defect and to confirm the watertight reconstruction. Once the bony borders of the defect were identified the surrounding mucosa was elevated. Meningoceles or meningoencephaloceles were reduced with bipolar forceps until the skull base level. Lyophilized fascia lata (TSF, Barcelona, Spain) was positioned “underlay” (between the bone and the dura) and covered with a free mucosal graft, usually harvested from the middle turbinate and occasionally from the inferior turbinate. At the cribriform plate, a true “underlay” technique is only feasible laterally. Thus, the fascia lata was positioned “onlay” (or “inlay”) and the medial aspect rotated towards the crista galli. The mucosal or mucoperiosteal graft from the turbinate is around 30% larger than the defect and once introduced it is surrounded by Surgicel or similar material to promote granulation tissue formation (Figures 3(a) and 3(b)). A finger cot packing is used at the end. No lumbar drainage was used in any of these defects.

## 3. Results

### 3.1. Group with Expanded Skull Base Surgery (*n* = 54)

This group included 66% female of mean age 47.7 ± 15.5, range 22–82 years.  Table 1 displays the different surgical approaches. A pedicled nasoseptal flap was harvested in 42 cases (78%) from the left side and the remnant from the right side. Five patients (9%) displayed clear symptoms of meningitis or outbreak postoperatively. In all an active CSF leak was evidenced and surgically repaired (3 patients with a pedicled nasoseptal flap and 2 with a pedicled rescue flap from the floor of the nose). During the follow-up of a mean of 15.6 ± 12.4 months (range 6–62 months) no recurrence of the CSF leak could be observed.

**Figure 4:**
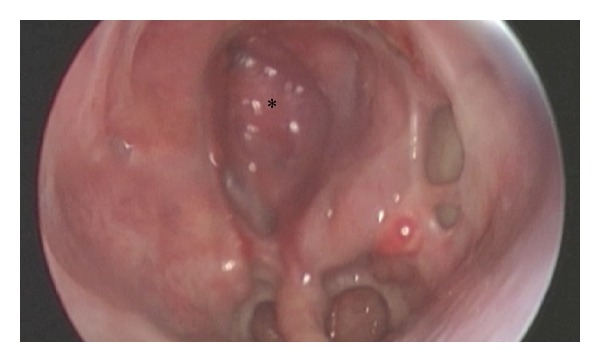
Same patient as in Figure 2(a) 4 months after reconstruction. Note the missing septum (vomer remnant inferiorly and the choanal border). *Reconstructed area of the nasoseptal flap.

**Figure 5:**
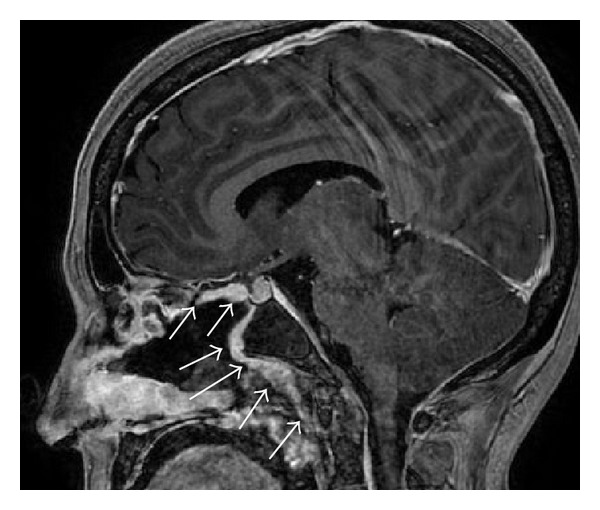
Sagittal view of the reconstructed planum sphenoidale, pituitary, and clivus. Note the enhancement of the perfusion of the pedicled nasoseptal flap (arrows).

Figures 4 and 5 show a postoperative result after skull base reconstruction with a pedicled nasoseptal flap.

### 3.2. Group with CSF Leaks of Other Origin (*n* = 62)

Sixty-two patients (52% women of mean age 48.8 ± 14.1, range 20–80 years) presented with a CSF leak, with unilateral watery rhinorrhea being the most frequent symptom. Five patients had a bilateral watery rhinorrhea as their leak was located in the sphenoid sinus (*n* = 4) or because of a septal perforation (*n* = 1). History of ascending bacterial meningitis was positive in 20 cases (32%), and 2 patients suffered from repeated meningitis. The most frequent aetiology was “spontaneous” in almost half of the study group, followed by trauma and surgery (Table 2). The cribriform plate concentrated half of the cases, followed by the ethmoid roof, sphenoid, and frontal sinuses (Table 3). The size of the defects ranged from 2 to 20 mm. Intrathecal application of sodium fluorescein was performed in all cases. Two patients developed temporary intense headaches and another one temporary weakness and paresthesia of the legs. The “underlay” reconstruction (material positioned between the dura and the bone of the anterior skull base) was used in 59/62 cases (95%) and the “inlay” technique (positioning of the material on the dura from within) in the other cases.

Closure of the leaks was accomplished in 61 patients (98.4%) after primary surgery. One patient presented with signs compatible with ascending bacterial meningitis two weeks after surgery. A persistent CSF leak was confirmed and the defect closed with a revision surgery. The follow-up at 75.3 ± 51.3 months (range 6–177 months) showed no evidence of CSF leaks recurrence.

## 4. Discussion

Skull base defects created during the removal of tumors are expected and therefore do not need further diagnostic procedures. However, the method and technique of reconstruction are the first question posed when planning expanded endoscopic approaches, as a permanent CSF leak may lead to ascending bacterial meningitis [2].

On the other hand, CSF leaks of other origin need to be investigated in depth. A thorough clinical history evaluating potential traumas, even long time before, prior to surgery or a history of bacterial meningitis is suspicious of an active or intermittent leakage. Occasionally, nasal endoscopy may reveal some pulsatile light reflex at the skull base or a soft tissue mass indicating a meningocele or a meningoencephalocele. However, when intracranial pressure is low or the leakage is intermittent endoscopy may be completely normal.

CAT scan in one millimeter slices allows a high resolution in the coronal and sagittal reconstruction. It is helpful in measuring the skull base defect radiologically and in planning the surgical technique of reconstruction. Rendering an exact picture of the bony framework is basic for the topographical diagnosis and the planning of the surgical approach. In cases in which a larger mass of tissue is seen in the CAT scan an additional MRI helps to assess meningoceles or meningoencephaloceles.

In active leaks a biochemical assessment with either beta-2 transferrin or beta-trace protein is helpful to differentiate from rhinorrhea due to chronic rhinosinusitis or to allergic rhinitis. In a literature review of 39 papers on the utility of testing beta-trace protein or beta-2 transferrin Bachmann-Harildstad could show that any are useful to assess the presence of CSF. Beta-trace has a high specificity and sensitivity, its results being faster (20 minutes versus 120 minutes of beta-2 transferrin) and less expensive [6].

Demarco et al. [7] used hypodense fluorescein, which seems to reduce the time of staining CSF down to 30 minutes.

Since its introduction in 1961 [8] intrathecal application of fluorescein has been shown to be very useful in the intraoperative assessment of the leakage and to prove the watertight closure. In 2 cases with suspicious intermittent leakage and negative biochemistry fluorescein was intrathecally applied as a diagnostic procedure. Both cases resulted in a negative result being excluded from this study. No serious complication could be observed after intrathecal fluorescein application. In all three cases in which headaches or weakness of the lower extremities was observed, the complaints were temporary leaving no sequelae. In an enquiry performed among rhinologists the habitual amount of fluorescein used was 0.5 and 1.0 mL at a concentration of 10%, although Senior et al. [9] could show effectiveness at low concentration and dosage of 0.1 mL at 10%. Complications after intrathecal fluorescein injection are usually related to increased dosages or concentrations or to a high administration speed [10, 11]. Severe complications (seizure, opisthotonus, and peripheric palsy) have been linked to chemical irritation in overdosage, as could be shown by Syms III et al. in an experimental study [12].

It is important to highlight that intrathecal application of fluorescein is an off-label indication and as such needs to be included specifically in the informed consent [10].

Among small and midsized defects spontaneous CSF leaks were the most frequent ones (48.4%) followed by those of a traumatic origin (24.2%). The cribriform plate was identified as the most frequent location (50%) followed by the anterior and posterior ethmoidal roof (32.3%). In a review of 55 papers including 1778 CSF leaks repaired endoscopically, the distribution between those of traumatic (50.2%) and nontraumatic origin (49.8%) was very similar [3]. Here too, spontaneous CSF leaks were the most frequent ones (41.1%) followed by those after endoscopic surgery (30.1%), trauma (23.2%) or tumor (5%), and congenital origin (3%).

The “underlay” reconstruction technique has been used the most (95%). Here, the dura is elevated in order to fit material, usually fascia lata, between the bone and the dura. In our hands, this is the preferred way to reconstruct the small and midsized skull base defects as the position of the fascia lata is stabilized by the underlying bone. However, in defects at the cribriform plate, the “underlay” position is difficult to achieve, as it would imply to fracture the intact cribriform plate when trying to detach the dura. This can be achieved laterally towards the ethmoid, but medially we tend to position the fascia in an L-shape mode towards the crista galli. Then, we wait for the intracranial pressure and pulsations to “push” the fascia from within until it adapts in an “inlay” position to the surrounding intact dura. For very small defects (2-3 mm) the “bath plug” technique with fat has proven reliable.

It is interesting to know that, in a meta-analysis of 289 CSF fistulas, Hegazy et al. [13] could show that different reconstruction techniques have a similar outcome. It looks as if any material used to reconstruct the skull base seems to work well.

The additional mucosal graft renders at least a two-layer reconstruction and protection of the dura. The size of both, fascia lata and mucosal graft, needs to be 2–5 mm larger than the defect itself. Particularly the latter has shown to shrink during the scarring process, as demonstrated by Hosemann et al. [14].

For the reconstruction of large skull base defects, specifically during expanded approaches, the pedicled nasoseptal flap has rendered spectacular rates of postoperative CSF leaks compared to the period before its description. The rate of postoperative CSF fistulas had been superior to 30%, compared to the 4% when using the nasoseptal flap [5]. The choice of the side from which to harvest the flap strongly depends on the tumor side, the presence of septal spurs or deviations, or the vascular compromise if drilling or surgery needs to be performed along the anterior wall of the sphenoid sinuses. Recent investigations have shown a negative impact of the flaps on olfaction, mucociliary transport, and quality of life [15–17]. Therefore, the cranial incision when harvesting the flap is nowadays situated below the area of the olfactory epithelium. Also, a reverse rescue flap from the contralateral side is performed to cover the denuded cartilage [18].

The reconstruction techniques between small/midsized and large defects cannot be compared. First, the location is a different one, with more spontaneous defects at the cribriform plate in Group 2; second, the extension of larger defects after expanded endoscopic surgery is usually linked to a high flow CSF leak. This indicates another type of reconstruction, including a lumbar drainage for the first days.

The use of lumbar drainage is still controversial. It is an invasive procedure, which may produce headaches, nausea, meningitis, or pneumoencephalus [19]. In an enquiry, Senior et al. found that 67% of the rhinologists in the States were routinely using a lumbar drainage in the management of CSF fistulas [9]. In a meta-analysis on 1568 CSF leaks, 761 lumbar drainages were used for 1 up to 10 days, although most studies showed that it was left for about 2–5 days. However, there is no way to calculate the potential benefit of a lumbar drainage due to the scarce data provided in the studies [3]. For small or midsized defects we do not see any indication. However, in larger defects, particularly those with a high pressure or high flow CSF leak, we believe that the area of reconstruction is better spared with a lumbar drainage as described above.

The prophylactic administration of antibiotics has not proved any effectivity in randomized studies, although we believe there is an indication for endoscopic skull base surgeries lasting 3–67 hours, as the nose cannot be considered sterile. Prophylactic antibiotics were administered routinely in 23 of 24 reviewed papers, in 4 studies only during the perioperative period and in 19 between 2 and 14 days, particularly in cases with lumbar drainage or nasal packing [3].

Hegazy et al. studied the results of 14 papers published between 1990 and 1999. The success rate varied between 60% and 100% (mean 90%) after primary surgery [13]. In a recent review of 55 papers dealing with endoscopic repair of CSF leaks, Psaltis et al. [3] confirmed the success rate of 90% for primary surgery and of 97% for rescue procedures with a complication rate lower than 0.03%. In their meta-analysis, Harvey et al. [20] conclude that skull base reconstruction with pedicled flaps renders a low postoperative CSF rate of 6.7% compared to a 15.6% after reconstruction with free grafts.

Endoscopic repair of CSF leaks is to be considered the gold standard for the majority of cases, as it is safe and effective [21]. The endoscopic closure rate in our groups was 91% after primary surgery for large skull base defects and 98% in small and midsized defects. After revision surgery the success rate was 100%. Demarco et al. [7] achieve an 88% closure rate at first attempt and 100% with a revision surgery, while Meco et al. obtained a 91% closure rate after primary surgery [22].

Interestingly, the rate of occult CSF fluid leakage after paranasal sinus surgery was found with beta-trace in two cases (2.9%) [23].

The weakness of our study is its retrospective design, the surgical outcome measurements based on a clinical follow-up only. However, the absence of both, an active CSF leakage and bacterial meningitis postoperatively, allows evaluating the rate of effective closure.

## 5. Conclusions

Considering all potential weaknesses of a retrospective study, we may confirm that endoscopic surgery for repair of either large, small, or midsized defects of the skull base seems to be a safe and effective procedure. Basically, any defect size can be reconstructed from within the nose. In our hands, the “underlay” technique with lyophilized fascia lata and a mucoperiosteal graft from mainly the middle turbinate achieved excellent results after primary surgery of smaller defects. No lumbar drainage is indicated here. However, the theoretical possibility of missed intermittent postoperative CSF leakage has not been extensively investigated in the present study.

Pedicled nasoseptal flaps are most adequate for the reconstruction of larger defects which happen after tumor removal. A temporary lumbar drainage seems to be helpful to reduce the intracranial pressure during the first days for a better scarring, although a prospective randomized study would be needed to give proof of its true benefit.

## Figures and Tables

**Table 1:** Type of skull base approach depending on the lesion.

Expanded endoscopic skull base approach	Final diagnosis
Transcribriform	Meningioma olfactory fossa (*n* = 2)
	Esthesioneuroblastoma (*n* = 2)
	Sinonasal carcinoma (*n* = 4)

Transtuberculum/transplanum	Meningioma planum sphenoidale (*n* = 5)
	Craniopharyngeoma (*n* = 6)
	Sarcoma (*n* = 1)
	Meningioma tuberculum sellae (*n* = 4)
	Rathke's cyst (*n* = 2)
	Suprasellar adenoma (*n* = 1)
	Polycystic astrocytoma (*n* = 1)

Transclival	Chordoma (*n* = 7)
	Chondrosarcoma (*n* = 3)
	Myxofibrosarcoma (*n* = 1)
	Fibrous dysplasia (*n* = 1)
	Inflammatory pseudotumor (*n* = 1)
	Petroclival meningioma (*n* = 3)
	Nasopharyngeal tumor x with extension to clivus (*n* = 2)
	Squamous cell carcinoma (*n* = 2)

Ethmoidal-pterigo-sphenoidal	Adenoma with extension to cavernous sinus (*n* = 3)
	Neurofibroma (*n* = 1)

Transorbital	Neurofibroma (*n* = 1)

Transpalatal	Congenital benign teratoma (*n* = 1)

**Table 2:** Etiology of CSF leaks.

Etiology	Number of cases (%)
Spontaneous	30 (48.4)
Traumatic	15 (24.2)
Iatrogenic/postoperative (FESS)	5 (8.1)
Benign tumor (osteoma, mucocele, and inverted papilloma)	5 (8.1)
Meningocele	3 (4.8)
Iatrogenic/postoperative (rhinoseptoplasty)	2 (3.2)
Congenital (meningoencephalocele of Sternberg's canal)	2 (3.2)

**Table 3:** Localizations of the CSF leaks.

Localization	Number of cases (%)
Cribriform plate	31 (50)
Anterior ethmoid	13 (21)
Posterior ethmoid	7 (11.3)
Sphenoid sinus	8 (12.9)
Frontal sinus	3 (4.8)

## References

[1] Bernal-Sprekelsen M, Bleda-Vázquez C, Carrau RL (2000). Ascending meningitis secondary to traumatic cerebrospinal fluid leaks. *The American Journal of Rhinology*.

[2] Jones NS, Becker DG (2001). Advances in the management of CSF leaks: new techniques will improve the management of unilateral clear nasal discharge. *British Medical Journal*.

[3] Psaltis AJ, Schlosser RJ, Banks CA, Yawn J, Soler ZM (2012). A systematic review of the endoscopic repair of cerebrospinal fluid leaks. *Otolaryngology—Head and Neck Surgery*.

[4] Hadad G, Bassagasteguy L, Carrau RL (2006). A novel reconstructive technique after endoscopic expanded endonasal approaches: vascular pedicle nasoseptal flap. *Laryngoscope*.

[5] Kassam AB, Thomas A, Carrau RL (2008). Endoscopic reconstruction of the cranial base using a pedicled nasoseptal flap. *Neurosurgery*.

[6] Bachmann-Harildstad G (2008). Diagnostic values of beta-2 transferrin and beta-trace protein as markers for cerebrospinal fluid fistula. *Rhinology*.

[7] Demarco RC, Tamashiro E, Valera FCP, Anselmo-Lima WT (2007). Use of a hypodense sodium fluorescein solution for the endoscopic repair of rhinogenic cerebrospinal fluid fistulae. *The American Journal of Rhinology*.

[8] KIRCHNER FR (1961). Use of fluorescein for the diagnosis and localization of cerebrospinal fluid fistulas. *Surgical forum*.

[9] Senior BA, Jafri K, Benninger M (2001). Safety and efficacy of endoscopic repair of CSF leaks and encephaloceles: a survey of the members of the american rhinologic society. *The American Journal of Rhinology*.

[10] Keerl R, Weber RK, Draf W, Wienke A, Schaefer SD (2004). Use of sodium fluorescein solution for detection of cerebrospinal fluid fistulas: an analysis of 420 administrations and reported complications in europe and the United States. *Laryngoscope*.

[11] Placantonakis DG, Tabaee A, Anand VK, Hiltzik D, Schwartz TH (2007). Safety of low-dose intrathecal fluorescein in endoscopic cranial base surgery. *Neurosurgery*.

[12] Syms CA, Syms MJ, Murphy TR, Massey SO (1997). Cerebrospinal fluid fistulae in a canine model. *Otolaryngology—Head and Neck Surgery*.

[13] Hegazy HM, Carrau RL, Snyderman CH, Kassam A, Zweig J (2000). Transnasal endoscopic repair of cerebrospinal fluid rhinorrhea: A0 meta- analysis. *Laryngoscope*.

[14] Hosemann W, Goede U, Sauer M (1999). Wound healing of mucosal autografts for frontal cerebrospinal fluid leaks—clinical and experimental investigations. *Rhinology*.

[15] Alobid I, Enseñat J, Mariño-Sánchez F (2013). Impairment of olfaction and mucociliary clearance after expanded endonasal approach using vascularized septal flap reconstruction for skull base tumors. *Neurosurgery*.

[16] Alobid I, Enseñat J, Mariño-Sánchez F (2013). Expanded endonasal approach using vascularised septal flap reconstruction for skull base tumors has a negative impact on sinonasal symptoms and quality of life. *The American Journal of Rhinology & Allergy*.

[17] de Almeida JR, Witterick IJ, Gullane PJ (2013). Quality of life instruments for skull base pathology: systematic review and methodologic appraisal. *Head and Neck*.

[18] Kasemsiri P, Carrau RL, Otto BA (2013). Reconstruction of the pedicled nasoseptal flap donor site with a contralateral reverse rotation flap: technical modifications and outcomes. *Laryngoscope*.

[19] Kerr JT, Chu FWK, Bayles SW (2005). Cerebrospinal fluid rhinorrhea: diagnosis and management. *Otolaryngologic Clinics of North America*.

[20] Harvey RJ, Parmar P, Sacks R, Zanation AM (2012). Endoscopic skull base reconstruction of large dural defects: a systematic review of published evidence. *Laryngoscope*.

[21] Komotar RJ, Starke RM, Raper DM, Anand VK, Schwartz TH (2013). Endoscopic endonasal versus open repair of anterior skull base CSF leak, meningocele, and encephalocele: a systematic review of outcomes. *Journal of Neurological Surgery A: Central European Neurosurgery*.

[22] Meco C, Arrer E, Oberascher G (2007). Efficacy of cerebrospinal fluid fistula repair: sensitive quality control using the beta-trace protein test. *The American Journal of Rhinology*.

[23] Bachmann G, Djenabi U, Jungehülsing M, Petereit H, Michel O (2002). Incidence of occult cerebrospinal fluid fistula during paranasal sinus surgery. *Archives of Otolaryngology—Head and Neck Surgery*.

